# HOXC6 Regulates the Epithelial-Mesenchymal Transition through the TGF-*β*/Smad Signaling Pathway and Predicts a Poor Prognosis in Glioblastoma

**DOI:** 10.1155/2022/8016102

**Published:** 2022-05-05

**Authors:** Sun Eryi, Li Zheng, Cai Honghua, Zhao Su, Xie Han, Pan Donggang, Zhou Zhou, Zhan Liping, Chen Bo

**Affiliations:** ^1^Department of Neurosurgery, Affiliated People's Hospital of Jiangsu University, Zhenjiang, Jiangsu Province 212002, China; ^2^Affiliated Hospital of Jiangsu University, Zhenjiang, Jiangsu Province 212002, China; ^3^Wujin Traditional Chinese Medicine Hospital, Changzhou, Jiangsu Province, China

## Abstract

**Background:**

The HOX gene family of transcription factors, characterized by conserved homeodomains, is positively correlated with the resistance to chemotherapy drugs and poor prognosis, as well as the initiating potential of gliomas. However, there are few studies regarding the HOXC6 gene in glioma cells. Therefore, in the present study, we explored the regulatory roles and detailed mechanisms underlying the relationship between HOXC6 and the progression of GBM.

**Methods:**

The expression levels and prognostic value of HOXC6 in GBM were evaluated using the data obtained from the GCCA, GEPIA, and ONCOMINE databases. The relationship between GBM prognosis and levels of HOXC6 was identified using Kaplan-Meier curves. The protein levels of HOXC6 in GBM and adjacent normal tissues were identified via Western blot and immunohistochemistry (IHC) staining methods. Lentiviruses containing full-length HOXC6 and HOXC6 specific siRNA sequences were used to overexpress and knock down, respectively, the expression of HOXC6 in U87 and U251 cells. The role of HOXC6 in the regulation of migration and proliferation of GBM cells was accessed using Transwell, wound healing, CCK-8, and colony formation assays. The activation of the TGF-*β*/Smad signaling pathway was detected via Western blotting.

**Results:**

Compared to normal tissues and control cells, GBM tissues and cell lines showed higher expressions of HOXC6. The expression of HOXC6 was associated with disease-free and the overall survival of GBM patients. Additionally, positive correlations between the expression of HOXC6 and the migration and proliferation of GBM cells were observed *in vitro*. The mechanistic analyses indicated that HOXC6 exerts its promotive effect on the progression and invasion of glioma cells by promoting the activation of the EMT and TGF-*β*/Smad signaling pathways.

**Conclusions:**

HOXC6 enhances the migration and proliferation of GBM by activating the EMT signaling pathway.

## 1. Introduction

Glioma is the most common tumor of the central nervous system (81% of all brain tumors). Additionally, glioblastoma (GBM) is considered the most aggressive type of glioma [1]. Based on the classification of nervous system tumors, low- and high-grade gliomas are defined as grades 1-2 and 3-4, respectively.

Regardless of the differentiation level, gliomas are characterized by infiltration, growth, and malignancy, leading to poor prognosis, high mortality, and a high possibility of recurrence. At present, the main treatments for glioma are microsurgery, radiotherapy, and chemotherapy [2]. However, the survival rate of GBM is very low among other malignant tumors. For example, its 5-year survival rate is under 5% [3]. Considering the gradual promotion of precision medicine in clinical practice, the lack of biologically active brain penetrating agent molecules and the insufficient understanding of the molecular characteristics of tumor genes hinder the application of targeted therapies. Hence, more effective gene-targeted therapies are very promising.

The HOX gene family of transcription factors, characterized by its highly conserved homeodomains, when overexpressed, can promote glioma's initiating potential, poor prognosis, and resistance to chemotherapy drugs. The HOXC gene is located at chromosome 12Q13.3 [4] and comprehends a subtype of the homeobox superfamily. Moreover, the HOXC6 gene is highly conserved and participates in many processes of embryonic development, cell morphogenesis, and differentiation regulation, including cell apoptosis, receptor signal transfer, differentiation, motility, and angiogenesis [5, 6]. The HOX family is also involved in the genesis, proliferation, migration, apoptosis, and epithelial-mesenchymal transformation of many tumor cells [7]. Therefore, these transcription factors can be used not only for the detection of tumor diseases and as a predictive index for tumor chemotherapy sensitivity [8] but also as a standard for evaluating the prognosis of patients [9]. It is also crucial to provide the basis for the use of HOX genes in targeted therapies. The HOXC6 gene plays an important role in breast cancer, prostate cancer, hematologic diseases, cervical cancer, and gastrointestinal tumors. Additionally, its abnormal expression has an important impact on various stages of tumor cell development [10–12]. However, there are few studies regarding the role of the HOXC6 gene in glioma cells, as well as in their occurrence and development.

The epithelial-mesenchymal transition (EMT) is a process that converts adherent epithelial cells into mesenchymal cells that can invade the extracellular matrix [13]. Moreover, metastasis and infinite proliferation are two important characteristics of tumor cells that can be promoted by the EMT. This transition has also been described as indispensable in embryo development and wound healing, also contributing to cell fibrosis, and cancer occurrence and development [14, 15]. The EMT promotion of the initiation, proliferation, metastasis, and drug resistance of tumor cells was previously identified by several recent studies [16]. Studies have also shown a significant association between the EMT and changes in cell polarity components, providing a new basis for the development of therapeutic targets to prevent tumor progression [17]. The EMT hallmark is the downregulation of E-cadherin, which enhances the instability of adherent junctions. However, the molecular mechanisms involved in the specific transformation process are complex, and in-depth studies are still being conducted [18]. The EMT can be induced by several growth factors produced by tumor-associated stroma, such as the transforming growth factor *β* (TGF-*β*), platelet-derived growth factor, epidermal growth factor, hepatocyte growth factor, and the heparin-binding growth factor [18]. In normal tissues, TGF-*β* acts as a tumor suppressor, while during tumorigenesis, it exerts tumor promotive activities. The TGF-*β* is also considered an inducer of receptor-regulated Smads (R-Smads) [19], thereby inhibiting the EMT by suppressing the activation of pathways related to TGF-*β*, which might comprehend a novel strategy to inhibit tumor progression.

In the present study, we demonstrated that the expression of HOXC6 was significantly increased in human GBM tissues and cell lines. Moreover, this higher expression of HOXC6 was positively correlated with the poor prognosis in GBM patients. The mechanistic analyses showed that the proliferation and migration of GBM cells were enhanced by HOXC6 through the activation of the mitogen-activated protein EMT pathway. Overall, our results indicated that HOXC6 can be a biomarker for GBM prognosis and a potential drug target for GBM treatments.

## 2. Materials and Methods

### 2.1. Bioinformatic Analyses

To evaluate the gene profiles of GBM patients, the datasets from TCGA Brain, Sun Brain, Murat Brain, Lee Brain, and Bredel Brain 2 were retrieved from ONCOMINE. The HOXC6-related gene expression datasets were retrieved from the TCGA database. For the gene expression analysis, a gene rank = top 10%, fold change = 1.5, and *p* value = 1*E* − 4 were set as the threshold [20]. The correlation between the expression of HOXC6 and the overall survival (OS) of GBM patients was analyzed using the GEPIA database [21]. The expression of HOXC6 in different pathological stages and the survival rate of patients were analyzed using the Chinese Glioma Genome Atlas (CGGA) database [22].

Gene set enrichment analysis (GSEA, http://www.broadinstitute.org/gsea/index.jsp) was used to detect genes sets of signaling pathways significantly different between higher and lower HOXC6 expression groups. The functional relationships between HOXC6 and other genes were tested by two-sided Pearson's product-moment correlations.

### 2.2. Construction of HOXC6 Knockdown

Two pairs of short hairpin RNAs (shRNAs) specifically targeting HOXC6 were designed for the lentivirus-mediated gene knockdown assay. The primer sequences were HOXC6 siRNA-1 forward: GUCCCUAUAACCAUCUAGUDTDT and reverse: ACUAGAUGGUUAUAGGGACDTDT and HOXC6 siRNA-2 forward: CCGUAUGACUAUGGAUCUADTDT and reverse: UAGAUCCAUAGUCAUACGGDTDT.

### 2.3. Quantitative Reverse Transcription PCR (qRT-PCR)

Total mRNA was extracted using TRIzol (Takara, A7603-1). The PrimeScript RT-PCR kit (Takara, PR036A-1) was used to synthesize cDNAs. The Bestar® Sybr Green qPCR master mix (DBI, DBI-2043) was used to detect the expression of indicated genes. All gene expression results were normalized to *β*-actin.

### 2.4. Human Samples and Clinical Information Collection

Human GBM samples (*n* = 24) and normal brain adjacent tissues (NBTs) were collected at the Zhenjiang First People's Hospital. No radiotherapy or chemotherapy was given before tissue collection. The OS was calculated based on the information collected from the date of diagnosis to the date of either death or last follow-up from 2019 to 2021 [23]. The NBTs were collected from patients with brain trauma. All procedures and usage of human samples followed the Declaration of Helsinki. All enrolled patients reviewed and signed written informed consent. All protocols and experimental designs were reviewed and approved by the Ethics Committee of the Zhenjiang First People's Hospital.

### 2.5. Cells and Cell Culture

The human U251 GBM cells were derived from our previous experiment and the U87 cell line from the Jiangsu University Affiliated Hospital. The A172, T98G, H4, and SHG44 cell lines were provided by the department of center laboratory Jiangsu Zhenjiang First People's Hospital.

### 2.6. Immunohistochemistry (IHC) Analyses

After being embedded in paraffin, the specimens were cut into 4 *μ*m sections. Then, they were deparaffined and rehydrated with xylene and different concentrations of ethanol, respectively. Sections were microwaved in antigen retrieval buffer (0.01 M citrate buffer, pH 6.0) for antigenic retrieval. Next, H_2_O_2_ (0.3%) was used to block the activity of endogenous peroxidases at room temperature for 15 min, and normal goat serum was used to reduce the nonspecific binding for 30 min. After overnight incubation with rabbit anti-HOXC6 polyclonal antibody (1 : 200, AB 252821, Abcam) at 4°C, tissues were washed three times with PBS. After washing, they were incubated for 1 h with indicated secondary antibodies. Then, samples were immersed, counterstained with Mayer's hematoxylin, and dehydrated. Sections were mounted using Crystal Mount and blindly evaluated by two pathologists. The staining score was as follows: no staining = 0, week staining = 1, moderate staining = 2, and strong staining = 3. Based on the ratio of positively stained areas to the whole area or entire section, we defined the staining extent as 1 (<25%), 2 (25–50%), 3 (50–75%), and 4 (75–100%). The percentage of tumor cells stained with HOXC6 and the score of staining intensity were represented as the staining index. Scores > 7.5 were identified as high HOXC6 expressions, and scores < 7.5 were identified as low HOXC6 expressions.

### 2.7. Measurement of Cell Proliferation

First, cells (3000 per well) were seeded in a 96-well plate incubated with the CCK-8 reagent (10 *μ*L) (LOT#GB707, Dojindo Molecular Technologies, Inc., Kumamoto, Japan) at 37°C for 4 h. An ELx800 plate reader (BioTek, Winooski, USA) was used to count cells by measuring the absorbance at 450 nm. The GraphPad Prism 7 software was used to statistically analyze the results. *p* < 0.05 was considered significantly different.

### 2.8. Colony Formation Assay

Cells (500 per well) were seeded in a 6-well plate and cultured for 14 days. After 14 days of culture, 4% paraformaldehyde and 0.5% crystal violet (C6158, Sigma-Aldrich, St. Louis, USA) were used to fix and stain the cells, respectively. The colonies were counted under a light microscope.

### 2.9. Wound Healing Assay

Cells (4.0 × 10^5^ per well) were seeded and incubated in a 6-well plate. After one day of culture, a scratching wound was generated using a 200 *μ*L pipette tip. A microscope with a camera was used to photograph the scratched region at indicated time points. Finally, the width of the scratch was calculated to evaluate the migration activity of cells.

### 2.10. Migration Assay

The Boyden chamber with a gelatin-coated polycarbonate filter (pore size = 8 *μ*m) was used to access the migrative activity of cells. Briefly, 2.0 × 10^6^ cells were seeded in the upper chamber, and 500 *μ*L 10% FBS medium was added to the lower chamber. After 24 h of culture, the cells that invaded the back of the membrane were stained using 0.1% crystal violet, followed by fixation with 4% PFA. Based on the total number of cells counted under the microscopy, the invasive capacity was calculated [23].

### 2.11. Western Blot

First, the RIPA buffer (Sigma) containing a protease inhibitor cocktail (Roche, USA) was used to lysate the indicated tissues and cells as previously described [24]. After measuring protein concentrations, 20 *μ*g of proteins was loaded and resolved using 10% SDS-PAGE gels. Then, protein bands were transferred to PVDF membranes, blocked with 5% fatty free milk for 1 h, and incubated with indicated primary antibodies for another two hours at RT or overnight at 4°C. After 3 times washing with PBST solution, proteins were incubated with indicated secondary antibodies at RT for 1 h. The WesternBright ECL Kits (Advansta, USA) and ChemiDoc™ XRS C imaging system (Bio-Rad) were used to detect and capture protein band images [24]. The primary antibodies (all 1 : 1000) used were E-cadherin (#ab231303, Abcam), vimentin (#ab137321, Abcam), TGF-*β*1 (SAB 41494), TGF*β*R2 (SAB 41495), HOXC6 (SAB 41032), p-Smad2 (#ab53100, Abcam), Smad2 (SAB 41442), and tubulin (Immunoway, YM3030).

### 2.12. *In Vivo* Experiments

Jiangsu University provided the BALB/c mice (6 weeks old). Subcutaneous injections of U87 siNC or U87-deficient siRNA1 were given to mice. Tumor volume measurements began two weeks after injection and were repeated every five days. The mice were killed after 27 d to determine the volume of the tumor, which was estimated using the formula: 1/2 × bigger diameter × smaller diameter^2^.

Tumors were paraffin-embedded after each trial [23]. The Institutional Animal Welfare Guidelines of the Chinese Academy of Medical Sciences authorized all animal care and research.

### 2.13. Statistical Analyses

The GraphPad Prism 7 and SPSS software were used for statistical analyses. The survival analysis was conducted using Kaplan-Meier curves and the Cox proportional hazards model. The log-rank test was carried out to determine the significance of variances between two groups. All results are represented as means ± SD. Each experiment was performed three times. *p* < 0.05 was defined as significantly different.

## 3. Results

### 3.1. High Expression of HOXC6 and Its Significant Correlation with Poor Prognosis of GBM Patients

**Figure 1:**
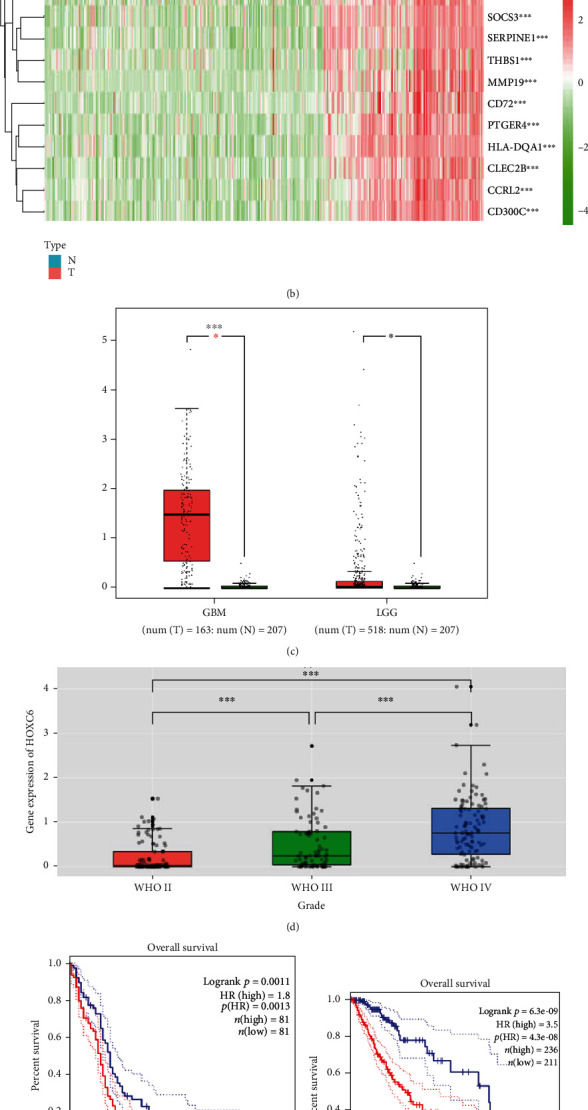
High expression and significant association of HOXC6 in GBM. The mRNA levels of HOXC6 were analyzed using the ONCOMINE gene profile database. In the images, the best gene rank percentile was used to define the color of the cells. The thresholds were gene rank = top 10%, fold change = 1.5, and *p* value = 1*E* − 4. (b) In the heat map of HOXC6 from the TCGA datasets, the topmost bar represents the sample type, in which blue represents normal control samples, and red represents tumor samples; the color gradation at the upper right represents gene expression, in which the color changes from red to green from the top to the bottom, indicating gene expression changes from high to low; the abscissa represents sample number, and the ordinate represents gene names; each rectangle corresponds to a sample expression value. The dendrogram on the left represents cluster analysis based on differences in gene expression. (c) HOXC6 was highly expressed in high- and low-grade gliomas in the GEPIA database. (d) HOXC6 expression data in the CGGA databases with high and low expressions of HOXC6. (e) The prognostic survival rates of GBM patients with low and high HOXC6 expressions were analyzed using the GEPIA database. (f) The prognostic survival rates of LGG GBM patients with low and high HOXC6 expression were analyzed using the GEPIA database. The prognostic survival rate of glioma patients with high HOXC6 expression was low, while the risk ratio was significantly higher (HR = 3.5; *p* = 4.3*E* − 8). (g) Using the CGGA database, we constructed Kaplan-Meier curves for patients with high and low HOXC6 expressions. ^∗^*p* < 0.05,  ^∗∗^*p* < 0.01, and^∗∗∗^*p* < 0.001. Data are shown as the mean ± SD.

Using the gene expression profiles of GBM patients downloaded from ONCOMINE, we showed that, compared to the normal healthy controls, HOXC6 expression was significantly increased in GBM patients (Figure 1(a)). These results were further confirmed using data from TCGA dataset of GBM patients (Figure 1(b)).

Then, we used the GEPIA database to identify the expression levels of HOXC6 in 163 high-grade gliomas (HGG, WHO: III-IV) and 207 normal brain tissues. We found that HOXC6 was expressed at high levels in HGG (*p* < 0.001, Figure 1(c)). We also used this database to analyze 518 low-grade gliomas (LGG, WHO I-II) and 207 normal brain tissues and showed that HOXC6 was still expressed at high levels in LGG (*p* < 0.05, Figure 1(c)). The data from the CGGA database confirmed the higher levels of HOXC6 in GBM samples compared to grade II and III samples (*p* < 0.001; Figure 1(d)).

Meanwhile, the survival rate of GBM patients with low or high HOXC6 expressions was analyzed using the GEPIA database and Kaplan-Meier curves. A total of 81 glioma patients with high HOXC6 expressions were compared to 81 glioma patients with low HOXC6 expressions. Compared to patients expressing low levels of HOXC6, patients with high expressions presented a low survival rate and high risk ratio (HR = 1.8; *p* = 0.0013; Figure 1(e)).

Then, we constructed Kaplan-Meier curves for LGG patients with high and low expressions of HOXC6. A total of 236 LGG patients were classified with high expression and 211 with low expression. The prognosis and survival rate of LGG patients with high HOXC6 expressions were significantly lower than those with low HOXC6 expression, and their risk ratio was also significantly higher (HR = 3.5; *p* = 4.3*E* − 8; Figure 1(f)).

Then, using the CGGA database, we constructed Kaplan-Meier curves for patients with high and low HOXC6 expressions. A total of 111 glioma patients with high expressions of HOXC6 were compared to 111 with low expressions. The prognosis and survival rate of glioma patients with high HOXC6 expressions were significantly lower than those with low expression (*p* < 0.0001; Figure 1(g)).

### 3.2. HOXC6 Is Highly Expressed in Human GBM Samples

**Figure 2:**
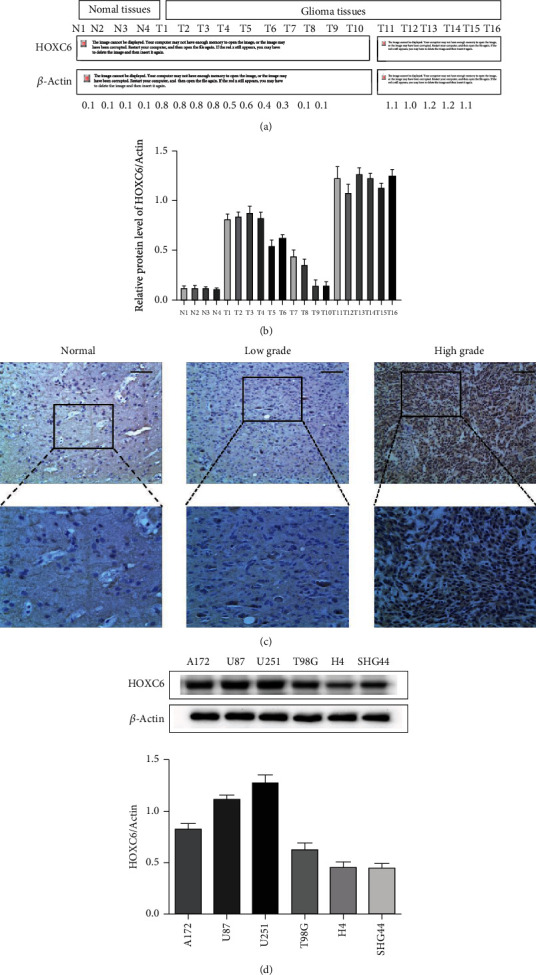
HOXC6 is highly expressed in human GBM samples. (a, b) Western blot results represent the protein levels of HOXC6 in indicated human tissues. (c) Representative images showing the expression of HOXC6 in noncancerous and glioma tissues measured by IHC. The scale bar represents 50 *μ*m. (d) The levels of HOXC6 in U251, U87, A172, T98G, H4, and SHG44 cell lines by Western blot.

Compared with the normal group, the protein levels of HOXC6 increased in the tumor groups, including groups T1, T2, T3, T4, T6, T7, and T11-T16 (Figures 2(a) and 2(b)). The protein levels of HOXC6 in other tumor groups were also higher than in the normal group, except for T9 and T10 samples (Figures 2(a) and 2(b)). To verify the expression of HOCX6 in GBM patients, NBT (*n* = 5) and GBM (*n* = 24) samples from the Zhenjiang First People's Hospital were analyzed using IHC. The expression of HOCX6 was identified in most GBM samples (21/24), but no expression was observed in normal control tissues (0/5) (Figure 2(c)). Based on the IHC results, eight cases showed relatively low HOCX6 expressions and 16 cases relatively high.

In the Western blot experiments, we evaluated the levels of HOXC6 in six glioma cell lines and found that the levels of HOXC6 increased to varying degrees. Thus, to select cell lines for the next experiment, the gray value calculation showed that the levels of HOXC6 in U251 and U87 glioma cells lines were significantly higher compared to A172, T98G, H4, and SHG44 cells (Figure 2(d)). Therefore, U251 and U87 cells were used in the subsequent experiments.

### 3.3. HOXC6 Promotes the Proliferation and Clonogenicity of GBM Cells

Furthermore, we used RNA interference (RNAi) to knock down HOXC6 in U251 and U87 glioma cells. The expression of HOXC6 was successfully downregulated, and the transfected cells were divided into three groups: siNC group, HOXC6 siRNA-1, and HOXC6 siRNA-2.

**Figure 3:**
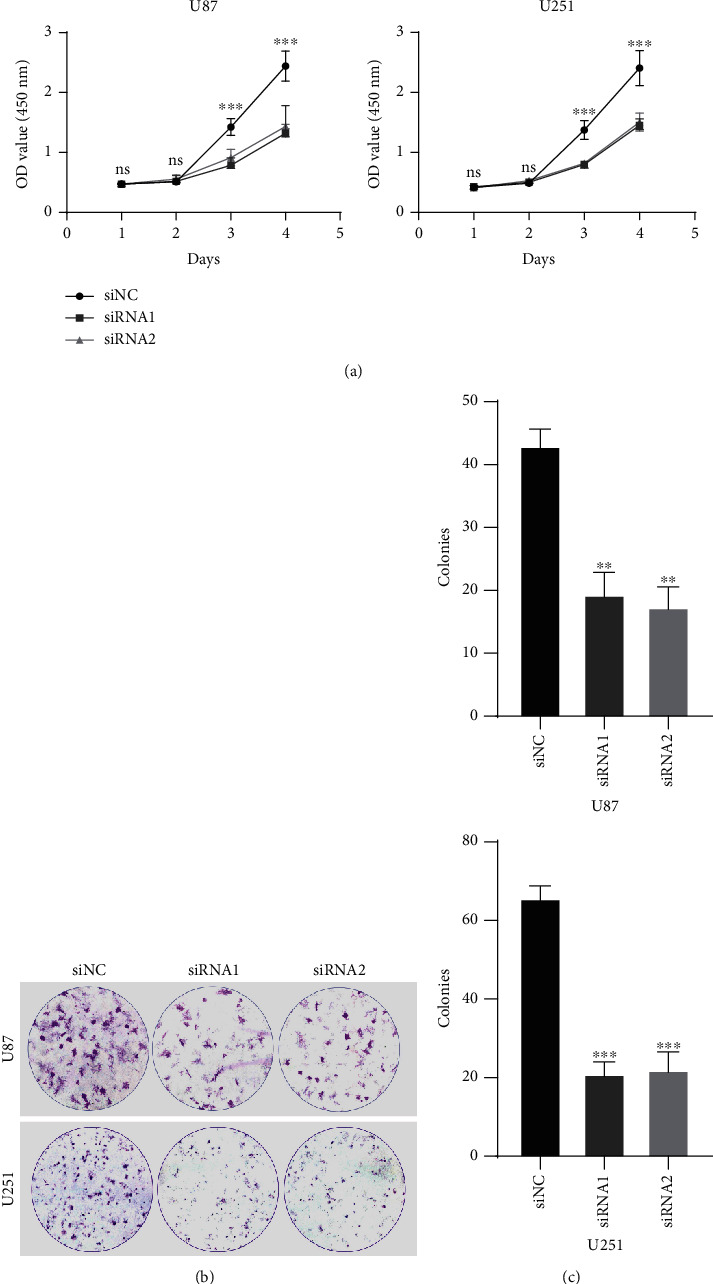
Effects of HOXC6 on the clonogenicity and proliferation of GBM cells. (a) The CCK-8 assay showed that HOXC6 inhibited the proliferation of glioma in U87 and U251 cell lines. (b, c) In the plate cloning experiment, the proliferation of U87 and U251 glioma cells in the siNC group was significantly higher compared to the siRNA1 and siRNA2 groups. ^∗^*p* < 0.05,  ^∗∗^*p* < 0.01, and^∗∗∗^*p* < 0.001. Data are shown as means ± SD.

Then, the inhibitory effect of HOXC6 on the proliferation of U251 and U87 glioma cells was observed by the CCK-8 assay. For U87 cells, the optical density (OD) of the three groups did not differ (*p* > 0.05) on days 1 and 2. Then, the OD of the siNC group significantly increased, and the HOXC6 siRNA-2 group increased faster than the HOXC6 siRNA-1 group. However, the OD of these two groups was similar and significantly lower compared to the siNC group (*p* < 0.001, Figure 3(a)).

The plate cloning assay indicated that the proliferation of HOXC6-deficient U87 and U251 cells, mediated by transfection with HOXC6-siRNA1 or HOXC6-siRNA2, was significantly impaired compared to the cells transfected with control siRNA (Figure 3(b)). Among the three groups of U87 and U251 glioma cells, the knockdown of HOXC6 using siRNA1 and siRNA2 suppressed cell proliferation compared to controls (*p* < 0.001, Figure 3(c)).

### 3.4. HOXC6 Promotes the Migration and Invasion of GBM Cells

**Figure 4:**
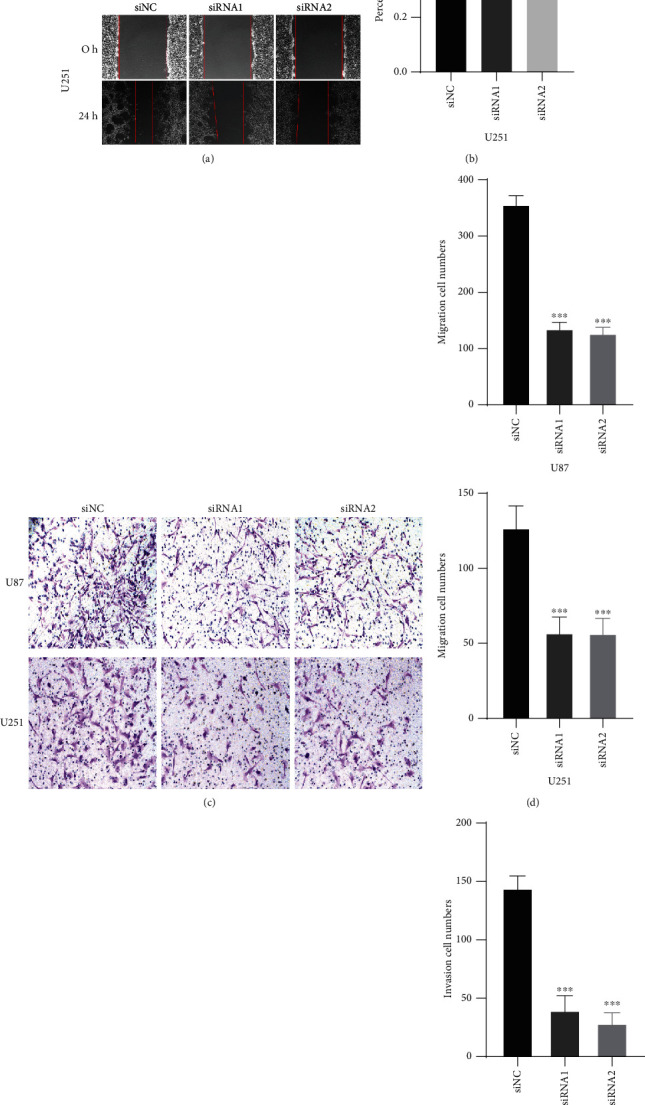
Effects of HOXC6 on the invasion and migration of GBM cells. (a, b) Images and bar graphs showing the migrative activity measured by the wound healing assay in control or HOXC6-deficient U87 and U251 cells. (c, d) The migrative activity of control or HOXC6-deficient U87 and U251 cells was also examined using the Transwell assay. (e, f) The invasive activity of control or HOXC6-deficient U87 and U251 cells was determined by the Transwell invasion experiment. ^∗∗∗^*p* < 0.01. Data are shown as means ± SD.

The migration of U251 and U87 glioma cells was inhibited after HOXC6 knockdowns. Compared to U251 and U87 cells transfected with siRNA-1 or siRNA-2, the migration ability of control cells (siNC) was significantly higher (*p* < 0.001, Figures 4(a) and 4(b)). In the Transwell cell migration experiment, the migration of glioma U87 and U251 cells decreased after HOCX6 expression was inhibited (Figure 4(c)). The number of cells in the U87 siNC group was 371.4 ± 18.61, comprehending an increase of 61.6 and 67.7% of the migration ability compared with the U87 siRNA1 (142.5 ± 10.17) and siRNA2 (119.8 ± 9.54) groups, respectively. The number of cells in the U251 siNC group was 127.6 ± 12.7, comprehending an increase of 66.8 and 68.3% compared to the U251 siRNA1 (42.4 ± 9.57) and siRNA2 (40.5 ± 8.57) groups, respectively. Overall, compared with the siRNA1 and siRNA2 groups, the number of cells in the siNC group was significantly increased as well as its migration ability (*p* < 0.001, Figure 4(d)).

In the Transwell chamber invasion experiment, the reduction of HOXC6 in U87 and U251 glioma cells was indicated by microscopic photographs (Figure 4(e)). The number of U87 cells in the siNC group was 159.3 ± 17.51, comprehending an increase of 70.7 and 76.9% in migration ability compared to the siRNA1 (46.7 ± 11.15) and siRNA2 (36.8 ± 8.44) groups, respectively. Moreover, the number of U251 cells in the siNC group was 39.1 ± 5.71, comprehending an increase of 72.4 and 67.5% compared to the siRNA1 (10.8 ± 2.14) and siRNA2 (12.7 ± 3.12) groups, respectively. Altogether, compared to control cells (siNC group), the knockdown of HOXC6 using HOXC6-siRNA1 or HOXC6-siRNA2 in U87 and U251 cells significantly impaired their proliferative and migrative capacity (*p* < 0.001, Figure 4(f)).

### 3.5. HOXC6 Participates in the Activation of the EMT Pathway

**Figure 5:**
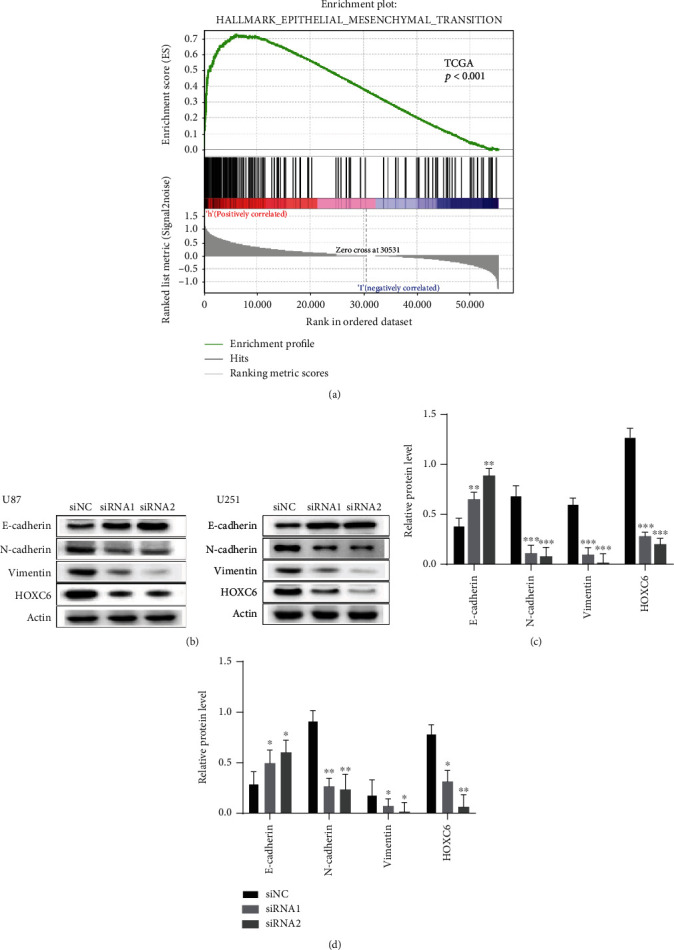
Association of HOXC6 with the EMT pathway. (a) The GSEA showed that the high expression of HOXC6 was correlated with enhanced expression of EMT signaling pathway components in the TCGA database. (b, c) Representative Western blot results showing the protein levels of vimentin, N-cadherin, E-cadherin, and HOXC6 in U87 and U251 cell lines and HOXC6-deficient GBM cells. ^∗^*p* < 0.05,  ^∗∗^*p* < 0.01, and^∗∗∗^*p* < 0.001. Data are shown as means ± SD.

The GSEA showed that high expression of HOXC6 was correlated with enhanced expression of EMT signaling pathway components in the TCGA database (Figure 5(a)). The expression of E-cadherin in the siRNA1 and siRNA2 groups was significantly upregulated compared to control cells (siNC group), while protein levels of N-cadherin were significantly downregulated in U87 glioma cells, as well as the protein levels of vimentin. Similar results were detected for U251 glioma cells (Figure 5(b)). According to the gray value calculation, the upregulation of E-cadherin protein levels in the siRNA1 and siRNA2 groups was statistically significant compared to the siNC group in U87 glioma cells (*p* < 0.05, Figure 5(c)). Similarly, the downregulation of N-cadherin and vimentin protein levels was statistically significant compared to controls in U87 glioma cells (*p* < 0.05, Figure 5(c)). Similar results were detected for U251 glioma cells (Figure 5(d)).

### 3.6. HOXC6 Activates the EMT through the TGF-*β*/Smad Pathway in GBM

**Figure 6:**
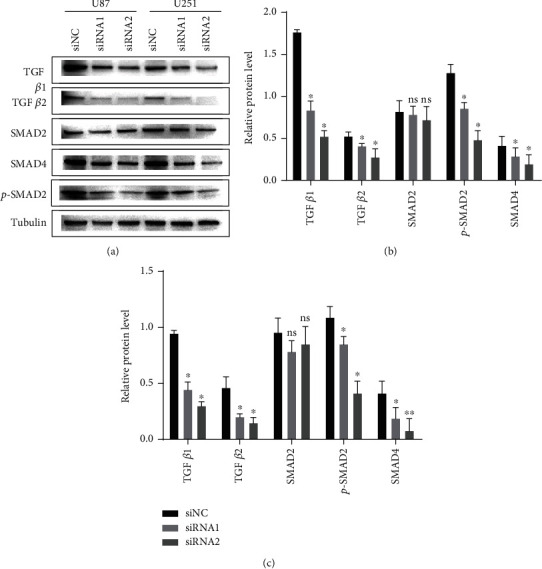
Activation of the EMT by HOXC6 in GBM. Expressions of TGF-*β*1, TGF-*β*2, p-Smad2, Smad4, and Smad2 in U87 and U251 cell lines and HOXC6 knockdown GBM cell models. ^∗^*p* < 0.05,  ^∗∗^*p* < 0.01, and^∗∗∗^*p* < 0.001. Data are shown as means ± SD.

The protein levels of TGF-*β*1, TGF-*β*2, Smad4, and p-Smad2 in the siRNA1 and siRNA2 groups were significantly downregulated compared to the siNC group in U87 glioma cells. On the other hand, the protein levels of Smad2 did not differ in U87 glioma cells compared to controls (siNC group) (Figure 6(a)). Similar results were detected for U251 cells (Figure 6(a)). According to the gray value calculation, the downregulation of TGF-*β*1, TGF-*β*2, Smad4, and p-Smad2 protein levels in the siRNA1 and siRNA2 groups was statistically significant compared to controls in U87 glioma cells (*p* < 0.05, Figure 6(b)). The protein levels of Smad2 did not differ in U87 glioma cells (*p* > 0.05, Figure 6(b)). Similar results were detected for U251 glioma cells (Figure 6(c)).

### 3.7. The Function of HOXC6 *In Vivo*

**Figure 7:**
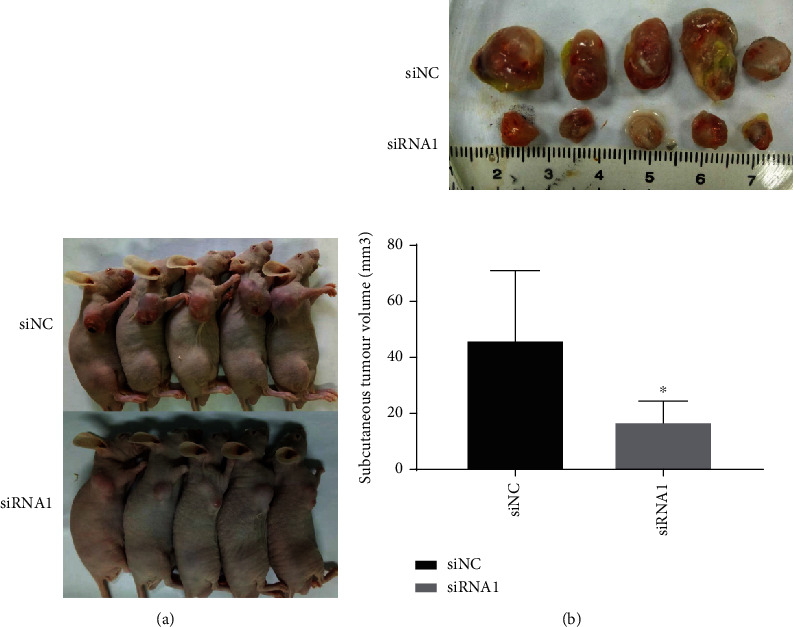
Function of HOXC6 *in vivo.* (a, b) Glioblastoma cells infected with HOXC6-deficient U87 siRNA1 or U87 control siNC. Representative pictures of subcutaneously implanted tumors and tumor volume quantification (b). ^∗^*p* < 0.05,  ^∗∗^*p* < 0.01, and^∗∗∗^*p* < 0.001. Data are shown as means ± SD.

The HOXC6 knockdown and control cells (U87 glioma cells) were intratumorally injected into a subcutaneous transplanted tumor model. HOXC6 knockdown suppressed glioblastoma cell growth *in vivo*, according to the tumor volume analyses (Figures 7(a) and 7(b)).

## 4. Discussion

Glioblastoma (GBM) is considered the most deadly and aggressive human cancer [25]. Moreover, the promotive effect of HOXC6 overexpression on the proliferative and migrative capacities of GBM cells was also observed by other researchers [20]. In the present study, we demonstrated that the poor prognosis of glioma patients was significantly and positively correlated with the HOXC6 expression. Then, we showed that HOXC6 exerts its promotive effect on the GBM cells' growth and migration by enhancing the EMT activation. Previous studies have shown increased expression of HOXC6 for multiple cancers, as well as its growth promotion ability in glioma cells [8, 20, 26]. Gene profile data obtained from ONCOMINE, TCGA, GEPIA, and CGGA databases have been previously used to analyze and verify the expression of HOXC6 in gliomas [3, 21] and the impact of increased HOXC6 expressions on the poor prognosis of glioma patients.

Here, we used data from GEPIA and CGGA and found that the expression of HOXC6 increased in both HGG and LGG patients and was significantly increased in GBM, which was also confirmed in the TCGA database. Then, the survival and prognosis of the GBM patients were analyzed. Based on the glioma database analyses, GBM patients with significantly increased HOXC6 expressions showed significantly lower prognosis and survival compared to those with relatively low HOXC6 expressions. Similar results were detected for LGG patients. Then, HGG and LGG cases were analyzed together, and we concluded that, in gliomas, the prognosis and survival rate significantly decreased in patients with high expressions of HOXC6. Overall, the expression levels of HOXC6 were significantly connected to patients' poor prognoses. Considering these results, we hypothesized that, in glioma tissue specimens, the expression of HOXC6 has a rising trend. Hence, we collected glioma specimens from patients in our hospital and performed protein and immunohistochemical analyses. The IHC results confirmed our hypothesis; that is, HOXC6 expression increased in high-grade glioma patients. Therefore, HOXC6 can be used to predict the outcomes of GBM patients.

Next, the protein levels of HOXC6 in human SHG44, H4, U87, A172, and U251 glioma cells were measured by Western blot. All cell lines presented increased expression of HOXC6. The lateral expression of HOXC6 was more pronounced and suitable in U251 and U87 cells. Thus, we used them in our subsequent experiments.

The poor prognosis and progression of tumors are mainly caused by their unlimited invasive and proliferative activity [27]. We hypothesized that the association between HOXC6 overexpression and poor prognosis was influenced by the promotion of glioma proliferation and migration. Therefore, we transfected U87 and U251 glioma cells with RNAi and knocked down HOXC6 to study its effects on the proliferative and migrative capacities of glioma cells. In the functional experiment, we showed that HOXC6 plays an important role in the proliferation and migration of gliomas. The proliferation and migration ability of U87 and U251 cells with HOXC6 knockdown were significantly reduced, which would be beneficial for the use of HOXC6 as a therapeutic target to improve the prognosis, survival rate, and quality of life of glioma patients. We also confirmed these results *in vivo*.

The EMT refers to the transformation of adherent epithelial cells into mesenchymal cells capable of invading the extracellular matrix. During the invasion of human cancer cells, tumor epithelial cells need to, at least temporarily, transform into the mesenchymal phenotype for invasion and metastasis. Therefore, the EMT is significantly connected with the metastasis, dissemination, and invasion of cancer cells [28]. Acquiring the ability to migrate and invade the extracellular matrix is considered a marker of EMT. Moreover, E-cadherin, N-cadherin, and vimentin also play important roles in tumor cells [29]. N-cadherin is a glycoprotein with a single-chain transmembrane domain that can regulate heterotypic and homotypic cell-cell adhesions and is involved in the regulation of the hematopoietic microenvironment, blood vessels, skeletal muscles, heart, brain, and nervous system [30]. N-cadherin is also considered an EMT marker since this process is characterized by the accumulation of mesenchymal markers, including N-cadherin, and reduced expression of E-cadherin. Thus, N-cadherin expression increases during the EMT [31]. We conducted a GSEA based on TCGA GBM databases and found that the EMT signaling pathway was enriched in the high HOXC6 expression groups. Then, the expression of E-cadherin in the siRNA1 and siRNA2 groups was significantly upregulated compared to controls, while N-cadherin and vimentin were downregulated in glioma cells. Therefore, compared to controls, the siRNA1 and siRNA2 groups presented inhibition of the EMT process. These results demonstrated that the downregulation of HOXC6 can affect the expression of related proteins during the EMT, indicating that this gene can also promote the invasive and migrative capacity of human U251 and U87 glioma cells through the EMT.

The TGF-*β* exerts its regulatory effects on GBM by controlling the activation of the EMT with both Smad-independent and Smad-dependent patterns, finally resulting in poor prognoses [32]. Previous studies have described enhancement of the E/N-cadherin transition, vimentin, and neurocadherin expressions, as well as attenuation of epithelial calmodulin expressions by TGF-*β* [33]. TGF-*β* combines with the receptors TGF-*β* RI/II to activate various downstream signaling pathways, including Smad, MAPK, and PI3K/Akt. Our current experiments demonstrated that HOXC6 can mediate both of these receptors. Among them, the TGF-*β*1/Smad pathway has been proved to mediate TGF-*β*-induced EMT. Additionally, the activation of types I and II serine-threonine kinase receptors, such as TbRI and TbRII, by TGF-*β*, results in the activation of R-Smads, and phosphorylation of Smad2 and/or Smad3, promoting the formation of heterotrimeric complexes with Smad4 and co-Smads [34]. After translocating into the nucleus, these complexes can enhance the expression of genes associated with EMT activation by interacting with different transcription factors. Thus, many transcription factors related to EMT activation are activated by Smad2/3 [35]. Here, we detected the expression of TGF-*β*1, TGF*β*R2, and the downstream molecules of the TGF-*β*1/Smad signaling pathway by Western blot. Besides, a predominant role of the Smad2/3 pathway in the activation of EMT induced by TGF-*β* in GBM cells was previously observed [18, 36]. The pathogenesis of cancers, including gliomas, can lead to dysregulation of TGF-*β* and its downstream Smad pathway [37, 38]. Herein, the activation of the TGF-*β*/Smad pathway and subsequent phosphorylation of Smad proteins were impaired by HOXC6 silencing, suggesting that HOXC6 exerts its regulatory effect on the migrative and invasive capacity of glioma cells by promoting the activation of the TGF-*β*/Smad pathway.

Our current study also has some limitations. First, a little bias might have been caused by the limited population enrolled. Thus, large sample sizes should be used in the future to verify our conclusions. Second, some oncogenes with aberrant expressions and other factors that also affect the survival of patients, such as adjuvant therapy, tumor differentiation, tumor stage, and age, were not incorporated in this study. Therefore, more comprehensive analyses containing this clinical information should be further performed to accurately evaluate the prognostic value of HOXC6 in GBM. Third, further *in vivo* investigations with animal models are required to verify the function of HOXC6 in GBM and reveal the exact molecular mechanism underlying the relationships between HOXC6 and the activation of the EMT pathway.

## Data Availability

Some or all data, models, or codes that support the findings of this study are available from the corresponding author upon reasonable request.
